# Double Trouble: The First Reported Case of Evans Syndrome Following RSV Vaccination

**DOI:** 10.3390/hematolrep17060068

**Published:** 2025-12-01

**Authors:** Mohammad Abu-Tineh, Deepika Beereddy, Ilse Ivonne Saldivar Ruiz, Divya Samat

**Affiliations:** 1 Department of Internal Medicine, Reading Hospital-Tower Health, West Reading, PA 19611, USA; divya.samat@towerhealth.org; 2 Department of Neurology, Reading Hospital-Tower Health, West Reading, PA 19611, USA; ilse.saldivarruiz@towerhealth.org

**Keywords:** Evans syndrome, RSV vaccine, cytopenia

## Abstract

**Background:** Evans syndrome is a rare autoimmune disease characterized by immune thrombocytopenia (ITP), autoimmune hemolytic anemia (AIHA), and autoimmune neutropenia, typically triggered by an episode of immune dysregulation or multiple other factors. We present what appears to be the first reported case of Evans syndrome developing in a 66-year-old female following respiratory syncytial virus (RSV) vaccination. **Case Presentation:** A 66-year-old female presented with a petechial rash on her arms, legs, and face. Laboratory tests revealed a platelet count of 1 × 10^9^/L, significantly lower than her historical baseline of >200 × 10^9^/L. On hospital day 4, her hemoglobin declined from 14.3 g/dL to 9.9 g/dL, with laboratory evidence of hemolysis, including elevated bilirubin, low haptoglobin, and increased lactate dehydrogenase (LDH). Bone marrow biopsy revealed megakaryocytic hyperplasia consistent with ITP, along with a small polyclonal B-cell population lacking CD20 expression. Imaging was unremarkable, showing no interval changes aside from stable pre-existing pulmonary nodules and no lymphadenopathy. These findings supported a diagnosis of Evans syndrome. Initial therapy with dexamethasone and intravenous immunoglobulin (IVIG) for presumed ITP was ineffective. Due to refractory thrombocytopenia, the patient initially received one dose of rituximab, followed by one dose of romiplostim. Subsequently, the patient received rituximab infusions every week at a rate of 375 mg/m^2^ for four doses, as well as prednisone at a dose of 1 mg/kg/day. Within five weeks, her blood count returned to normal. **Conclusions:** This case raises concern for a potential temporal association between RSV vaccination and the onset of Evans syndrome. It underscores the need for heightened clinical awareness and further investigation into immune-mediated hematologic complications following RSV immunization.

## 1. Introduction

In the absence of another cause, Evans syndrome is a rare, chronic autoimmune disorder characterized by the simultaneous or sequential occurrence of ITP and AIHA and, less frequently, autoimmune neutropenia. The syndrome can be primary or secondary, with secondary cases often linked to primary immunodeficiencies, systemic autoimmune diseases, lymphoproliferative disorders, or, less commonly, exposure to certain drugs or vaccines [1,2]. Recent studies have estimated that the incidence was 1.8 per million person–years for adults and between 0.5 and 1.2 per million person–years for children. With five-year survival rates as low as 38% for adults and higher mortality rates in children, the prognosis remains poor, especially in secondary cases [2,3]. Pathogenesis studies suggest that an underlying immune dysregulation model, characterized by a high frequency of pathogenic variants in immune-regulatory genes, particularly in cases with pediatric onset, highlights the complexity of Evans syndrome’s pathogenesis [4,5]. Treatment is not yet fully standardized, instead being based on expert consensus; however, the most common first-line treatment is corticosteroids, and the second-line treatment is commonly rituximab [1].

After receiving the influenza and mRNA COVID-19 vaccine, reports have been made of vaccine-induced immune cytopenia, including ITP and, in rare instances, Evans syndrome [6,7,8,9,10,11]. With extensive post-marketing surveillance of RSV vaccines, data showed no increased risk of ITP and only a slight excess risk of Guillain–Barré syndrome [12,13,14]. Previously, Evans syndrome had not been reported as an adverse event following RSV vaccination [12]. The introduction of RSV protein subunit vaccines has nevertheless been a significant advancement in preventing severe RSV disease in older adults [10]. To date, there have been no published reports that systematically describe Evans syndrome following the administration of the respiratory syncytial virus vaccine [12,13]. The lack of documented cases highlights a gap in our understanding of possible autoimmune hematologic complications that can occur after RSV vaccination [12,13]. This case report widens the range of possible immune-mediated hematologic complications after immunization by describing a new instance of Evans syndrome temporally associated with RSV vaccination [6,7,8,9,10,11,12,13].

## 2. Case Presentation

A 66-year-old female with a past medical history of hypertension, generalized anxiety disorder, hyperlipidemia, gastroesophageal reflux disease, and obstructive sleep apnea presented to the Emergency Department with an urgent care referral for a new-onset petechial rash involving her arms, legs, and face.

The initial laboratory investigation showed a platelet count of 2 × 10^9^/L, urging immediate transfer to the ED. Her previous laboratory tests showed normal platelet levels. The patient denied any history of recent infections, fever, recent travel, sick contacts, prior history of platelet disorders, known autoimmune disease, or family history of platelet or bleeding disorders. Her home medications included amlodipine, losartan, hydrochlorothiazide, and escitalopram. She reported receiving the RSV vaccine one week before admission.

On presentation, her vital signs were stable. Laboratory workup showed a white blood cell (WBC) count of 5.4 × 10^9^/L, a hemoglobin level of 14.3 g/dL, and a severely reduced platelet count of 1 × 10^9^/L, with a normal chemistry panel. HIV and hepatitis panels were all negative.

Initially, the patient was started on expectant treatment for presumed ITP with high-dose dexamethasone 40 mg daily for 4 days (days 1–4) and a 1 g/kg dose of intravenous immunoglobulin (IVIG) on days 2–3. On hospital day 4, her hemoglobin declined from 14.3 g/dL on admission to 11.6 g/dL, prompting the initiation of hemolysis testing, including lactate dehydrogenase (LDH), haptoglobin, direct antiglobulin (Coombs) test, and a peripheral smear, all of which had not been obtained on admission, given the initially normal hemoglobin. These day-4 studies revealed a sudden rise in total bilirubin, low haptoglobin, elevated LDH, and a positive direct antiglobulin test for IgG (C3d negative), consistent with hemolytic anemia. A detailed timeline of the patient’s clinical course is summarized in Table 1.

Further serologic testing showed an ANA titer of 1:80 with a cytoplasmic pattern and 1:40 with a nuclear homogenous pattern, findings considered low-level and nonspecific, but suggestive of underlying immune dysregulation consistent with Evans syndrome. No additional autoimmune workup was pursued. CMV IgG positive, positive EBV IgG, and negative IgM. Cardiolipin IgG < 2.0, Cardiolipin IgM < 2.0. The hematopathology study did not provide definitive immunophenotypic evidence of a hematolymphoid neoplasm. The bone marrow biopsy revealed a hypercellular marrow with 65% cellularity, an increased immature megakaryocyte fraction without clustering, a myeloid-to-erythroid ratio of approximately 2:1 with 34% erythroid precursors showing full maturation, ~14% red blood cell precursors by flow cytometry, preserved granulocytic maturation, absence of increased blasts, dysplasia, or clonal lymphoid populations, and a normal karyotype findings most consistent with normal to mildly increased erythropoiesis.

CT scans from 2021 to 2025 consistently demonstrated stable, scattered, and tiny pulmonary nodules without any interval change. Most recently, these nodules were characterized as benign, with no suspicious features and no evidence of lymphadenopathy.

**Figure 1:**
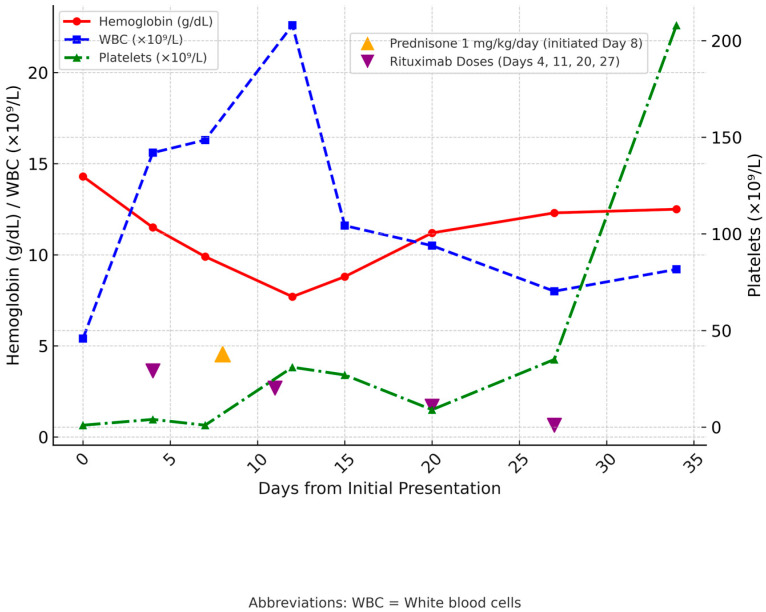
WBC, Hemoglobin, and Platelet Trends from Presentation to Clinical Endpoint. The *x*-axis shows days from initial presentation. The left *y*-axis depicts hemoglobin (g/dL) and white blood cell counts (×10^9^/L), while the right *y*-axis shows platelet counts (×10^9^/L). Vertical lines indicate therapeutic interventions: prednisone 1 mg/kg/day was initiated on day 8, and rituximab was administered on days 4, 11, 20, and 27. Abbreviations: WBC = White blood cells.

**Figure 2:**
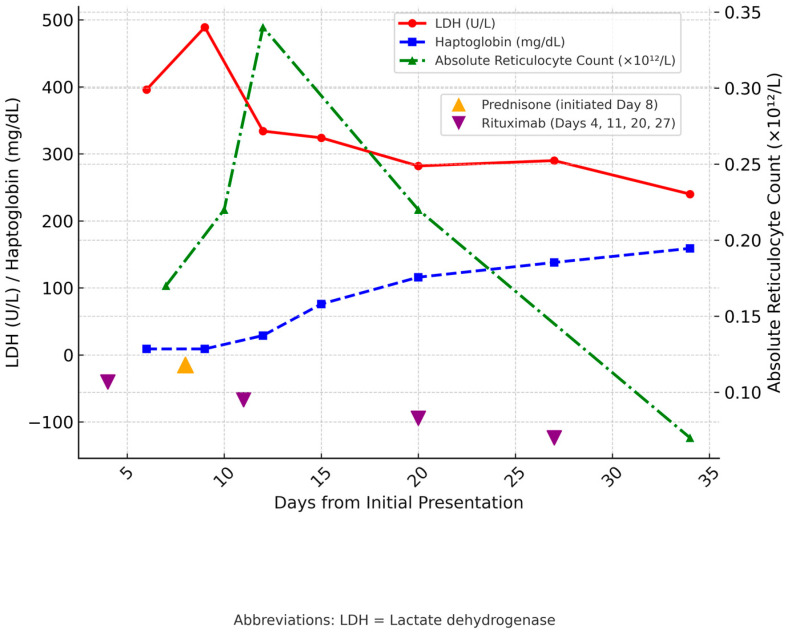
Trends of Haptoglobin, LDH, and Absolute Reticulocyte Count Over Time. The *x*-axis shows days from initial presentation. The left *y*-axis depicts LDH (U/L) and haptoglobin (mg/dL), while the right *y*-axis shows the absolute reticulocyte count (×10^12^/L). Interventions are indicated by markers on the *x*-axis: an upward orange triangle represents prednisone initiated on Day 8, and downward purple triangles represent rituximab doses administered on Days 4, 11, 20, and 27. Abbreviations: LDH = Lactate dehydrogenase.

Due to refractory thrombocytopenia, the patient initially received one dose of rituximab, followed by one dose of romiplostim. Afterward, she continued on rituximab infusions every week at a rate of 375 mg/m^2^ for four doses, along with prednisone at 1 mg/kg/day. Within five weeks, her blood count returned to normal. She had required IVIG only once on admission and did not undergo blood type or crossmatch, as no transfusion was ever needed. Notably, there was no recurrence of hemolysis during the remainder of her clinical course. Figure 1 and Figure 2 illustrate the temporal trends in hematologic parameters and hemolytic markers, respectively, during the clinical course and response to immunosuppressive therapy.

## 3. Discussion and Conclusions

To the best of our knowledge, this is the first case documented of Evans syndrome secondary to RSV vaccination. The clinical hospital course, lack of alternative etiologies, and temporal association are consistent with cases of vaccine-associated Evans syndrome reported after receiving other vaccinations, such as influenza and COVID-19 [6,7,8,9,10,11]. The elevated prevalence of damaging variation in immune-regulatory genes in pediatric cohorts supports the pathophysiological mechanism, which is thought to involve aberrant activation of autoreactive lymphocytes in genetically or immunologically susceptible individuals [4,5]. Furthermore, Evans syndrome is increasingly understood as a clinical manifestation of underlying immune dysregulation, characterized by aberrant T- and B-cell responses, expansion of circulating T-follicular helper cells, chronic T-cell activation, and reduced naïve CD4+ T cells, regardless of whether a genetic defect is identified [14]. RSV infection and vaccination can further trigger Th2/Th17 polarization, excessive cytokine production, and impaired interferon signaling, contributing to immunopathology and potentially precipitating autoimmune cytopenia in genetically or immunologically susceptible individuals [15,16,17]. Host genetic variation plays a decisive role in modulating immune responses and disease severity in RSV infection and related complications [18,19].

In pediatric Evans syndrome, up to 65% of cases harbor damaging variants in immune-regulatory genes, including LRBA, CTLA4, and STAT3, which are associated with more severe, treatment-refractory disease and increased risk of additional immunopathologic manifestations [4,5,20,21]. Systematic genetic testing is recommended in pediatric patients with Evans syndrome to guide prognosis and enable targeted therapy. In contrast, in adults, testing should be considered selectively based on early onset, family history, or atypical features [22]. Autoantibody panels and immunophenotyping should be considered in patients with Evans syndrome secondary to RSV vaccination, particularly in cases that are severe or refractory. These investigations can identify underlying immune dysregulation, with common findings including hypogammaglobulinemia, lymphoproliferation, and abnormal lymphocyte subsets [4,14,20]. Early identification of immune dysregulation is critical for prognosis and management, as patients with definable immune dysregulation are more likely to require multiple lines of immunomodulatory therapy and have a more severe disease course [17]. However, the utility of these investigations in adult-onset Evans syndrome and specifically in the context of RSV vaccination requires further research.

Rarely, autoimmune cytopenia can develop as a result of the immune system’s reaction to vaccination, especially when adjuvanted protein subunit vaccines are used. In the literature, seven cases of Evans syndrome have been identified following vaccination [6,7,8,9,10,11] (Table 2).

Despite widespread vaccination, the sporadic occurrence of Evans syndrome—while biologically plausible—underscores the overall safety of RSV vaccines. In a cohort of more than 4.7 million older adults, a slight increase in Guillain–Barré syndrome was observed; however, no association with Evans syndrome was identified, and no excess risk of ITP was reported [23]. Evans syndrome has not been recognized as a vaccine-related adverse event in early safety data from clinical trials and post-licensure analyses [13,23]. Knowing the risk–benefit of the vaccine, the risk of immune-mediated hematologic complications remains very low compared with the benefits of vaccination in preventing severe RSV disease in high-risk populations [23,24].

Usually, a poor prognosis is expected in Evans syndrome, given the high morbidity and death from infections or bleeding, and the possible underlying associated malignancy [2]. Immunosuppressive treatment must be recognized and started as soon as possible. In refractory cases, rituximab is the recommended second-line treatment, while corticosteroids are the first-line treatment. Evans syndrome ideally requires multidisciplinary care and long-term monitoring due to its chronic, relapsing nature [1]. This case highlights the importance of ongoing pharmacovigilance and educating physicians about immune-mediated hematologic complications that can occur post-vaccination, which are rare but potentially severe. Additionally, it highlights the importance of customized risk assessment, particularly for patients with known or suspected immune dysregulation. Nevertheless, given their significant benefit in lowering RSV-related morbidity and mortality, the overall evidence favors the continued use of RSV vaccines in eligible populations [12,13,24]. A five-year follow-up study conducted in 2021 within an Italian military cohort assessed the safety of repeated immunizations, covering tetanus, diphtheria, polio, meningococcal polysaccharide, and hepatitis A vaccines. The investigators found no evidence of late-onset autoimmune or lymphoproliferative disorders, confirming the long-term safety of multiple vaccinations. Significantly, protective antibody levels against the administered vaccines remained elevated throughout the follow-up period, with sustained seroprotection evident in the majority of subjects. No indications of B-cell polyclonal activation or other measures of immune dysregulation were observed, even in individuals who had received multiple vaccines simultaneously. These results reinforce that routine immunizations, even when given in combination, do not trigger chronic immune pathology. In this context, Evans syndrome after RSV vaccination should be considered an atypical and unexpected event rather than an expected outcome of extensive vaccination exposure [25]. Further research is necessary to better understand the immunogenetic risk factors that contribute to these adverse events and to inform future vaccine safety monitoring and risk-mitigation strategies.

## Figures and Tables

**Table 1:** Clinical Timeline of events from presentation to hematologic remission.

Time Point	Clinical Event	Diagnostic Workup	Treatment & Outcome
Day 0–1	Presented to ED with petechial rash on arms, legs, and face	CBC: Platelets 1 × 10^9^/L, Hb 14 g/dL	Dexamethasone 40 mg/day and IVIG started: suspected vaccine-induced ITP
Day 4	Still inpatient	Platelets 4 × 10^9^/L, Hb 11.6 g/dL	Four doses of dexamethasone, two doses of IVIG completed: 1st Rituximab dose given
Day 6–7	Clinical decline noted	Hb 9 g/dL, ↑ LDH, ↓ Haptoglobin, ↑ Bilirubin; Coombs IGG positive; ADAMTS13 pending	Evans syndrome diagnosed; Romiplostim 1 mcg/kg given.
Day 8	Persistent thrombocytopenia	Platelets 2 × 10^9^/L, Hb 9.8 g/dL; CT: negative; Bone marrow & flow cytometry done	Prednisone 1 mg/kg/day (90 mg/day)
Day 11	Ongoing anemia and low platelets	Platelets 16 × 10^9^/L, Hb 7.9 g/dL	2nd Rituximab dose administered
Day 12	Discharged from the hospital	Platelets 31 × 10^9^/L, Hb 7.7 g/dL, WBC 22.6; Haptoglobin 29 mg/dL, LDH 334 U/L; BM biopsy: ITP	Prednisone 60 mg/day; planned two more Rituximab doses, outpatient
Week 3	Outpatient follow-up	Platelets 9 × 10^9^/L, Hb 11.2 g/dL, WBC 10.5 × 10^9^/L	3rd Rituximab dose: Prednisone increased to 80 mg/day
Week 4	Outpatient follow-up	Platelets 35 × 10^9^/L, Hb 12.3 g/dL, WBC 8 × 10^9^/L	4th Rituximab dose: Prednisone 80 mg/day was continued
Week 5	Outpatient follow-up	Platelets 208 × 10^9^/L, Hb 12.5 g/dL, WBC 9.2 × 10^9^/L	Hematologic remission achieved; 12-week steroid taper planned

Abbreviations: ED = Emergency Department; CBC = Complete blood count; WBC = White blood cells; Hb = Hemoglobin; LDH = Lactate dehydrogenase; ITP = Immune thrombocytopenia; IVIG = Intravenous immunoglobulin; CT = Computed tomography; BM = Bone marrow. Symbols: ↑ Elevated, ↓ Low.

**Table 2:** Previously reported cases of Evans syndrome post vaccination.

Vaccine Type	Author/Year	Onset	Hemoglobin (g/dL)	Platelets (×10^9^/L)	Direct Coombs	Key Treatments
COVID-19 mRNA (BNT162b2, Pfizer)	Hidaka et al., 2022 [6]	≈2 weeks after 2nd dose	6.9	39	Positive (also indirect+)	Prednisolone ~1 mg/kg/day; RBC transfusion
COVID-19 mRNA (BNT162b2, Pfizer)	Cvetković et al., 2023 [8]	8 days after 2nd dose	4.5	27	Positive (IgG+++)	Prednisone; azathioprine; dexamethasone; IVIG; RBC transfusions
COVID-19 mRNA (BNT162b2, Pfizer/Comirnaty)	De Felice et al., 2022 [9]	≈7 days post-vaccination	10.0	8	Positive (3+)	Methylprednisolone; IVIG ×5; rituximab; eltrombopag; prednisone
COVID-19 adenoviral vector (ChAdOx1, AstraZeneca)	Gambichler et al., 2022 [10]	≈2 weeks post-vaccination	9.9	1	Positive (warm anti-IgG)	High-dose prednisolone; dexamethasone; IVIG
COVID-19 mRNA (BNT162b2, Pfizer)	Ng et al., 2023 [11]	≈1 week after 2nd dose	5.8	7	Positive	Pulse IV methylprednisolone; IVIG; rituximab
Influenza (seasonal)	Shlamovitz & Johar, J Emerg Med 2013 [7]	4 days post-vaccine	-	<5	Positive direct Coombs	Oral prednisone + IVIG

## Data Availability

The original contributions presented in this study are included in the article. Further inquiries can be directed to the corresponding author(s).

## Abbreviations

ITP	Immune Thrombocytopenic Purpura
AIHA	Autoimmune Hemolytic Anemia
BM	Bone Marrow
CT	Computed Tomography
LDH	Lactate Dehydrogenase
